# Non-Motor Symptoms and Health-Related Quality of Life in Patients with Isolated Dystonia: A Cross-Sectional Study

**DOI:** 10.3390/healthcare13151824

**Published:** 2025-07-26

**Authors:** Ovidiu Lucian Băjenaru, Lidia Băjenaru, Alexandru Balog, Alexandru Constantinescu, Octavian Andronic, Cătălina Raluca Nuță

**Affiliations:** 1 Faculty of Medicine, “Carol Davila” University of Medicine and Pharmacy, 050474 Bucharest, Romania; ovidiu.bajenaru@umfcd.ro (O.L.B.); alexandru.constantinescu@umfcd.ro (A.C.); octavian.andronic@umfcd.ro (O.A.); catalina.nuta@umfcd.ro (C.R.N.); 2 National Institute of Gerontology and Geriatrics “Ana Aslan”, 11241 Bucharest, Romania; 3 Department of Communications, Applications, and Digital System, National Institute for Research and Development in Informatics—ICI Bucharest, 011455 Bucharest, Romania; 4 Department of Computer Science, Faculty of Automatic Control and Computers, National University of Science and Technology POLITEHNICA Bucharest, 060042 Bucharest, Romania; 5 Doctoral School of Economic Informatics, Bucharest University of Economics Studies, 010374 Bucharest, Romania; sandu.balog@gmail.com; 6 Gastroenterology Department, University Emergency Hospital, 050098 Bucharest, Romania; 7 General Surgery Department, University Emergency Hospital of Bucharest, 050098 Bucharest, Romania

**Keywords:** dystonia, non-motor symptoms (NMS), health-related quality of life (HRQoL), Short Form Health Survey (SF-36), Patient Health Questionnaire-9 (PHQ-9), Montreal Cognitive Assessment (MoCA)

## Abstract

**Background/Objectives:** Dystonia, traditionally regarded as a purely motor disorder, is now increasingly recognized as involving clinically significant non-motor symptoms (NMSs) that can adversely affect patients’ health-related quality of life (HRQoL). This study aimed to assess HRQoL in Romanian patients with isolated dystonia and to evaluate the impact of two key NMSs, depression and cognitive impairment, on their HRQoL. We hypothesized that depression would have a greater adverse effect on HRQoL than cognitive impairment. **Methods**: A cross-sectional study was conducted involving 65 adult Romanian patients with isolated dystonia. HRQoL was measured using the Short Form-36 Health Survey (SF-36), including the physical component summary (PCS) and mental component summary (MCS). Depressive symptoms were assessed using the Patient Health Questionnaire-9 (PHQ-9), and cognitive impairment was assessed using the Montreal Cognitive Assessment (MoCA). Descriptive statistics, correlation analysis, and parametric and non-parametric tests were used. Multiple regression analysis was employed to evaluate associations between NMS and HRQoL. **Results**: The mean (SD) age was 56.6 (14.3) years, and 80% of participants were female. Depression and cognitive function were significantly associated with PCS (0.33 and −0.51, respectively) and MCS (0.26 and −0.78, respectively). Multiple regression analysis showed that the two NMS explained 38% of the variance in PCS and 58% of the variance in MCS. Depression had a greater impact on PCS and MCS than cognitive impairment (−0.47 vs. 0.33 and −0.72 vs. 0.16, respectively). Cognitive impairment (MoCA < 26) was present in 35.4% of patients, while 46.2% had at least mild depressive symptoms (PHQ-9 ≥ 5); 23.1% met criteria for moderate-to-severe depression (PHQ-9 ≥ 10). Depressive symptoms showed strong negative correlations with all SF-36 domains, while cognitive performance correlated modestly. **Conclusions:** Both depression and cognitive impairment have a significant negative impact on HRQoL in dystonia, with depression having a stronger effect, as we hypothesized. Routine screening for non-motor symptoms is essential to support better clinical outcomes and enhance patients’ quality of life.

## 1. Introduction

Dystonia is a chronic, often disabling hyperkinetic movement disorder characterized by sustained or intermittent involuntary muscle contractions, resulting in abnormal postures and repetitive movements [1]. Prevalence estimates for isolated dystonia vary, with newer studies suggesting that it may be more common than previously believed. A large-scale epidemiological study reported a prevalence of 35.1 per 100,000 individuals [2], contrasting with earlier global estimates of around 16.4 per 100,000 [3], which may have underestimated the true prevalence of the disease due to underdiagnosis and limited awareness of its diverse presentations.

Once considered purely a disorder of motor circuits, it is now evident that primary (idiopathic) dystonia has important non-motor dimensions, including psychiatric, cognitive, sensory, and sleep-related symptoms, which contribute significantly to its overall clinical manifestation [4,5]. These non-motor manifestations, such as depression, anxiety, cognitive impairment, and sensory disturbances, are increasingly recognized as integral to the clinical profile of dystonia and have significant implications for patients’ quality of life [6,7].

A series of studies has highlighted the critical role of non-motor symptoms (NMS) in dystonia. These symptoms may precede or accompany motor symptoms and often have a greater impact on patients’ quality of life than motor dysfunction alone [6,7,8,9]. These insights have driven a paradigm shift toward multidimensional, patient-centred assessments that acknowledge the full clinical complexity of dystonia.

Dystonia is becoming more recognized from a pathophysiological standpoint as a network disorder that affects the cerebello-thalamo-cortical and cortico-striatal circuits in addition to the basal ganglia. Both motor and non-motor regulation, including mood and cognition, are influenced by these networks. Depression, anxiety, and cognitive impairments in dystonia have all been linked to altered inhibitory control, dopaminergic and serotonergic dysfunction, and maladaptive plasticity within these circuits. The significant impact of non-motor symptoms on quality of life may be explained by these mechanisms [10,11].

Despite a growing body of research, few studies have specifically investigated the impact of dystonia on health-related quality of life (HRQoL). Even fewer have examined how NMS collectively affects HRQoL in these patients. Depressive symptoms have consistently been identified as a major determinant of HRQoL in patients with dystonia [6,8], whereas cognitive function has been less thoroughly investigated [12,13,14].

The Short Form-36 Health Survey (SF-36) is one of the most widely used generic tools for measuring HRQoL [6]. SF-36 [15] is extensively applied in clinical practice, epidemiological studies, and health policy research. In addition, several tools are widely used in NMS research, such as the Patient Health Questionnaire-9 (PHQ-9) to detect depressive symptoms [16], and the Montreal Cognitive Assessment (MoCA) to screen for cognitive impairment [17].

Studies carried out in diverse international settings using the SF-36 have shown that patients with different forms of dystonia often experience reduced health-related quality of life, particularly in the presence of depression or cognitive dysfunction [18,19,20,21,22,23,24].

Although large-scale epidemiological data on adult-onset isolated focal dystonia (AOIFD) are currently limited in Romania, preliminary clinical research suggests that individuals affected by AOIFD may experience notable physical, emotional, and social challenges [9,25,26,27]. The SF-36, PHQ-9, and MoCA scales have each been validated in Romanian clinical populations. For example, the SF-36 has been used in studies involving patients undergoing haemodialysis [28], as well as those with osteoporosis [29], diabetes [30], and spinal muscular atrophy [31]. The PHQ-9 has shown strong reliability in Romanian populations with diabetes [32] and among young females [33]. The MoCA has also been validated in patients with mild cognitive impairment [34].

The SF-36, PHQ-9, and MoCA scales have each been validated in Romanian populations. However, no prior study has combined these instruments to examine how non-motor symptoms relate to HRQoL in dystonia, particularly in Romania. Therefore, the primary goal of this study was to assess HRQoL in Romanian patients with isolated dystonia and to evaluate the impact of depressive symptoms and cognitive impairment on HRQoL. We hypothesized that both depression and cognitive impairment would be associated with lower HRQoL, with depression having a more pronounced effect than cognitive impairment.

## 2. Materials and Methods

### 2.1. Study Design

This cross-sectional study was conducted at the Neurology Department of Colentina Clinical Hospital in Bucharest, a tertiary referral centre and a national leader in botulinum toxin therapy for movement disorders. A cross-sectional design was chosen as an efficient initial approach to explore associations between HRQoL and non-motor symptoms in this patient population. A total of 131 patients with dystonia were consecutively recruited between November 2017 and January 2020. Diagnoses were confirmed by board-certified neurologists using internationally accepted clinical diagnostic criteria [26]. The STROBE (Strengthening the Reporting of Observational Studies in Epidemiology) guidelines were followed in the reporting of this cross-sectional observational study. Health-related quality of life (HRQoL), as measured using the SF-36 instrument, was the main outcome, and all variables were measured at a single time point.

Inclusion criteria were: (a) adult-onset idiopathic focal dystonia; (b) self-identified Romanian ethnicity; and (c) ability to provide informed consent and complete the assessment tools in Romanian.

Exclusion criteria included: (a) generalized, segmental, or multifocal dystonia at onset; (b) secondary dystonia due to structural, metabolic, or pharmacological causes; (c) genetically confirmed primary dystonia; and (d) major psychiatric or systemic comorbidities that could bias cognitive or quality-of-life assessments. The use of antidepressant medication was neither assessed nor controlled for in this study. These criteria were applied to ensure diagnostic specificity and minimize potential confounding factors in the evaluation of non-motor symptoms.

Of the 131 enrolled patients, 65 were selected for a comprehensive assessment of non-motor symptoms (MoCA, PHQ-9, and SF-36), based on their availability for extended visits and ability to complete the full evaluation protocol during routine follow-up appointments. All 65 patients completed the entire set of questionnaires; therefore, no data were missing from the analyses. Standardized clinical and demographic data were collected and stored in an anonymized database. For this study, no formal a priori power analysis was carried out. The final sample size of 65 participants, however, is consistent with empirical guidelines for performing multiple linear regression analyses with a small number of predictors, given its exploratory and cross-sectional design. The stable estimation of parameters in preliminary models requires at least 10 to 15 observations per predictor, according to established methodological guidance. The sample size was considered appropriate for identifying preliminary associations without risking the integrity of the model, since each regression model contained two predictors (PHQ-9 and MoCA) [35]. The study was approved by the Ethics Committee of Colentina Clinical Hospital, and all participants gave written informed consent prior to enrolment.

### 2.2. Measures

Based on their demonstrated validity, clinical applicability, and prior use in both general neurology and dystonia research, the PHQ-9, MoCA, and SF-36 were chosen. These instruments have shown strong psychometric qualities in a variety of demographics and focus on three key constructs that are pertinent to the goals of the study: cognitive functioning, health-related quality of life, and depression symptoms. RAND’s licensing terms for academic research use were followed when using the validated Romanian version (Version 1.0) of the SF-36.

#### 2.2.1. Demographic and Clinical Variables

The following demographic variables were selected for this study: sex, age at survey, and education. Sex was classified as female or male. The age at survey was considered a continuous variable and a categorical variable (age groups) with four age categories: equal to or less than 44 years, 45–54 years, 55–64 years, and 65 years or older. Education was grouped into two categories: ≤12 years and >12 years.

Regarding clinical variables, we selected age at onset and body distribution, according to methodological recommendations detailed in [1]. The age at onset reported by patients was considered a continuous variable and a categorical variable. It was classified into two groups: equal to or less than 40 years (early adulthood) and greater than 40 years (late adulthood). Body distribution was divided into focal (involving a single body region) and segmental (affecting two or more contiguous body parts).

In addition, the disease duration (years) was calculated for this study from the difference between the age at survey and the self-reported age at onset of disease. It was considered a continuous variable and a categorical variable. It was classified into two groups: equal to or less than 10 years and greater than 10 years. Additionally, the type of dystonia was grouped into three categories: cervical dystonia, blepharospasm, and other types (hemifacial spasm, writer’s cramp).

#### 2.2.2. Health-Related Quality of Life

HRQoL was measured using the Romanian version 1 of the SF-36, a validated instrument developed by Ware et al. to assess overall health status across physical and mental dimensions [15]. The questionnaire comprises 36 items, with 35 contributing to eight subscales that reflect key aspects of health: physical functioning (PF), role limitations due to physical problems (RP), bodily pain (BP), general health perceptions (GH), vitality (VT), social functioning (SF), role limitations due to emotional problems (RE), and mental health (MH). One additional item evaluates self-perceived change in health over time (health transition). Each subscale score is transformed to a 0–100 scale, where higher scores indicate better perceived health status and quality of life. These subscale scores can be presented as a health profile or combined into two summary measures: the Physical Component Summary (PCS) and the Mental Component Summary (MCS), offering a global evaluation of physical and mental health. Scoring procedures followed the guidelines provided by the developers [15].

Briefly, the scoring procedure consists of the following steps [15,36]. First, all eight subscale scores (range = 0–100) are standardized to the relevant population using a linear z-score transformation. Second, z-scores are multiplied by the subscale factor score coefficients for PCS and MCS and summed over all eight subscales. Scoring is normed using published means, standard deviations, and aggregated weights. The norm-based scores allow for comparing scores between different populations or health conditions. Previous studies used either orthogonal or oblique rotation to derive country-specific scores for the SF-36 [15,36,37,38]. Finally, the PCS and MCS scores are transformed so that each has a mean value of 50 and a standard deviation of 10 (T-score) in the total sample. A T-score above 50 indicates better health-related quality of life, compared to the total norm population. Norm-based scoring is recommended for easier interpretation [15].

In this study, the mean and standard deviation from the US general population [15] were used to standardize the SF-36 scales, and the corresponding factor weight coefficients for component summary scores (from an obliquely rotated solution [36,37]) were applied for the US norm population to facilitate international comparisons.

#### 2.2.3. Non-Motor Symptoms

Depressive symptoms were assessed using the PHQ-9, a widely used, self-administered instrument based on DSM-IV criteria for major depressive disorder [16]. Participants rated the frequency of symptoms over the previous two weeks on a 4-point scale from 0 (“Not at all”) to 3 (“Nearly every day”), resulting in a total score ranging from 0 to 27. Interpretation followed standard cut-offs: 0–4 (no depression), 5–9 (mild), 10–14 (moderate), 15–19 (moderately severe), and 20–27 (severe depression). PHQ-9 scores were analysed both continuously and categorically, with participants classified as either having no depressive symptoms (0–4) or exhibiting any level of depression (5–27).

Cognitive function was evaluated using MoCA, a validated screening tool for detecting mild cognitive impairment across a wide range of clinical populations. The MoCA covers seven core cognitive domains—visuospatial and executive functions, naming, memory, attention, language, abstraction, delayed recall, and orientation—through 16 items, yielding a maximum score of 30 points. A score of 26 or higher was considered normal, while scores below 26 indicated cognitive impairment, further categorized as mild (18–25), moderate (10–17), or severe (<10). For statistical analyses, MoCA scores were used both as continuous variables and dichotomized, with participants grouped into those with normal cognitive function (≥26) and those with impairment (<26).

The primary outcomes were the Physical Component Summary (PCS) and Mental Component Summary (MCS) scores derived from the SF-36. Predictor variables of interest were the PHQ-9 total score (depressive symptoms) and the MoCA score (cognitive function).

### 2.3. Data Analysis

Descriptive statistics were used to describe and summarize the data. Categorical variables were reported as frequencies and percentages, and their group differences were assessed by the Chi-square test or Fisher’s exact test. Continuous variables were reported as the mean (M) with standard deviation (SD) and median (Mdn) with interquartile range (IQR). Spearman’s correlation coefficient was calculated to evaluate the relationship between the continuous variables.

Prior to conducting the statistical analyses, the normality of continuous variables was evaluated using the standard errors of skewness and kurtosis, complemented by visual inspection of histograms and Q–Q plots. These assessments indicated that some variables deviated from normality, particularly within smaller subgroups.

Consequently, group differences in outcome measures were examined using either parametric tests (independent t-tests or one-way ANOVA) or non-parametric alternatives (Mann–Whitney U or Kruskal–Wallis H), depending on the distribution of the data and the results of Levene’s test for homogeneity of variances.

Bivariate analyses were then conducted to explore associations between MoCA, PHQ-9, and SF-36 scores and demographic or clinical variables.

The main relationships between depressive symptoms, cognitive function, and HRQoL were further evaluated using multiple linear regression models. Preliminary checks confirmed that the assumptions of linearity, normality of residuals, and homoscedasticity were not violated for these models.

Age and sex were not included as covariates in the final regression models. Although these variables were collected and analysed descriptively and in bivariate tests, no significant associations were found between them and either the main predictors (PHQ-9 and MoCA) or the outcome variables (PCS and MCS). Furthermore, their inclusion did not meaningfully alter the estimated effects of depressive symptoms or cognitive function. To preserve model parsimony and reduce the risk of overfitting given the sample size, we excluded these demographic variables. This approach is consistent with recommended practices in exploratory cross-sectional research, where model simplicity and empirical relevance guide covariate selection [39,40,41].

A statistical analysis was conducted using IBM SPSS Statistics software, version 24.0, with the significance level set at *p* < 0.05 (two-tailed).

## 3. Results

### 3.1. Demographic and Clinical Characteristics

Table 1 provides an overview of patients’ characteristics for the total sample and stratified by sex. Out of a total of 65 patients, 52 (80%) were female, and the mean age was 56.6 years (SD = 14.3; range: 27–82 years). The number of years of education ranged from 7 to 16 years, with a mean of 12.86 (SD = 2.28). The mean age of onset was 48.3 years (SD = 13.1). Most patients (71%) had focal dystonia and the mean disease duration was 8.2 years (SD = 7.4; range: 0–34 years).

### 3.2. Health-Related Quality of Life (HRQoL)

Descriptive statistics of the SF-36 domains and summary measures are presented in Table 2. All eight SF-36 scales were scored on a 0 (worst) to 100 (best) scale. The two summary measures (PCS and MCS) are T-scores (mean = 50, standard deviation = 10) calculated against the US reference population. In the total sample, the highest mean score was recorded on the social functioning domain (84.3), followed by the role of emotional factors (77.4). The lowest mean score was for the role of the physical domain (51.3), followed by the bodily pain domain (57.9). The mean MCS score was higher than the mean PCS score, with values of 46.2 and 43.5, respectively. Notably, 61.5% (n = 40) of the patients with dystonia scored below average in the PCS. Similarly, 53.8% (n = 35) of the patients scored below average on the MCS.

Spearman’s correlation coefficients among the eight scales and between the eight scales and the PCS and MCS were calculated (Table 3). All correlations were statistically significant (*p* < 0.05), except the correlation between RP and GH (*p* > 0.05). Correlations among SF-36 subscales that were statistically significant ranged from 0.31 (BP with RE and MH, GH with RE) to 0.78 (VT with SF).

As shown in Table 3, the correlations between the PF, RP, BP domains and the PCS measure are higher (ranging from 0.73 to 0.81) than the correlations of these domains with the MCS measure (ranging from 0.50 to 0.65). At the same time, the correlations between the VT, SF, RE, and MH domains and the MCS are higher (from 0.62 to 0.89) than the correlations of these domains with the PCS (from 0.47 to 0.66). The GH domain has noteworthy correlations with both measures, but slightly higher with the MCS than with the PCS (0.65 vs. 0.63). Among the eight SF-36 dimensions, RP was the best measure of physical health, and MH was the best measure of mental health. In contrast, RP and BP were the poorest measures of mental health, and RE was the poorest measure of physical health. The correlation between PCS and MCS was strong (0.77).

Table 4 and Table 5 report the results of the bivariate analysis performed between all SF- 36 domains (including summary measures) and the demographic and clinical variables. In all eight domains of SF-36, male patients had higher mean scores than female patients, but this difference was statistically significant only for the PF (*p* = 0.003), RP (*p* = 0.033), and VT (*p* = 0.033). In the total sample, male patients had significantly higher PCS and MCS mean scores than female patients (*p* = 0.008 and *p* = 0.036, respectively). The BP domain had statistically significantly higher mean scores in patients under 44 years of age (*p* = 0.029). Additionally, the GH domain had statistically significantly higher mean scores in patients under 44 years (*p* = 0.003), in those with more than 12 years of education (*p* = 0.039) and in those with dystonia onset before age 40 years (*p* = 0.005).

Patients with focal dystonia had significantly higher mean scores than patients with segmental dystonia in PCS (*p* = 0.014) and in the following physical domains: PF (*p* = 0.028), RP (*p* = 0.028), and GH (*p* = 0.028). Patients with shorter disease duration (≤10 years) had significantly higher mean scores than patients with higher disease duration in PCS and MCS (*p* = 0.046 and *p* = 0.034, respectively), as well as in PF (*p* = 0.040), RP (*p* = 0.019), and SF (*p* = 0.046). No other differences were statistically significant.

### 3.3. Non-Motor Symptoms: Descriptive Findings

The overall mean was 5.95 (SD = 5.39; range: 0 to 22) for depression and 26.17 (SD = 2.85; range: 17 to 30) for cognitive impairment (Table 6), with a statistically significant negative correlation between the two subscales of −0.25 (*p* = 0.041). No significant differences were found between female and male patients in MoCA and PHQ-9 scores (*p* > 0.05).

As shown in Table 7, all included patients had depression symptoms of varying severity according to the PHQ-9 scale. Notably, 46.2% of patients scored ≥5 on the PHQ-9 (at least mild depressive symptoms), and 23.1% scored ≥10 (moderate or greater depressive symptoms). Cognitive impairment was also present in 35.4% of the patients.

Depressive symptoms and cognitive impairment showed no significant association with any of the demographic or clinical variables selected for this study (*p* > 0.05), except for the association between cognitive impairment and body distribution, which was statistically significant (*p* = 0.038). 

### 3.4. Associations of HRQoL with Depressive Symptoms and Cognitive Impairment

Spearman correlation coefficients were calculated between SF-36 measures (the eight scales, PCS and MCS) and non-motor symptoms measured using PHQ-9 and MoCA (Table 8). The PHQ-9 score was negatively correlated with the eight SF-36 domains and statistically significant (*p* < 0.05 in all cases). The highest correlation was with the mental health domain (−0.76). Nevertheless, the correlation between the PHQ-9 and the MCS was higher than the correlation with the PCS (−0.78 vs. −0.51).

As shown in Table 8, all correlations between the MoCA score and the eight SF-36 domains were positive, but only those with the physical functioning, physical role, and bodily pain domains reached statistical significance (*p* < 0.05). The highest correlation was with the bodily pain domain (0.35). The correlation between MoCA and PCS was 0.33 (*p* < −0.01), which was higher than the correlation between MoCA and MCS (0.26, *p* < −0.05). Generally, correlations are stronger for depression than for cognitive impairment.

To study the non-motor symptoms related to PCS and MCS and to investigate which non-motor symptoms mainly contributed to lower HRQoL, we performed the multiple regression analysis. Two models were constructed in which the PCS and MCS were dependent variables, and the independent variables included in each of the two models were PHQ-9 and MoCA (Table 9).

In the first analysis, the results showed a significant model (R^2^_adj_ = 0.376, *p* < 0.001), which PCS associated with the MoCA score (β = 0.334, *t* = 3.312, *p* = 0.002), and PHQ-9 score (β = −0.471, *t* = −4.678, *p* < 0.001). In the second analysis, the results showed a significant model (R^2^_adj_ = 0.584, *p* < 0.001), which associated the MCS score with the MoCA score (β = 0.542, *t* = 2.009, *p* = 0.049), and PHQ-9 score (β = −0.723, t = −8.796, *p* < 0.001). In the first model, the MoCA and PHQ-9 explained 37.6% of the variance in PCS, and, in the second model, 58.4% of the variance in MCS. The remaining variance is attributable to other variables not included in the models.

Finally, we determined how the different levels of depressive symptoms and cognitive impairment were associated with HRQoL. For this purpose, patients were divided as having normal (MoCA score ≥ 26) or abnormal (MoCA score < 26) cognitive function, as well as having minimal (PHQ-9 score < 5) or mild, moderate to severe (PHQ-9 score ≥ 5) depressive symptoms. Table 10 reports the bivariate analysis performed between the SF-36 measures (the eight domains and the two summary measures) and scoring categories for non-motor symptoms (the MoCA and PHQ-9).

As expected, PCS scores were higher in the group of patients without cognitive impairment (*p* = 0.040) and the group of patients without depression (*p* < 0.001). MCS scores were also higher in the group of patients without depressive symptoms (*p <* 0.001). Although the MCS of patients without cognitive impairment was higher than that of patients with cognitive impairment, the difference was not statistically significant (*p* = 0.349). It is worth noting that all differences in scores in the eight domains of the SF-36 between the groups of patients without depression and with depression were statistically significant (*p* < 0.05). The scores of each domain of the SF-36 were higher in the group of patients without depression. The scores of the SF-36 domains were higher in the group of patients without cognitive impairment than in the group of patients with cognitive impairment, but statistically significant only in the physical role (*p* = 0.015) and bodily pain (*p* = 0.013).

## 4. Discussion

This cross-sectional study focused on the HRQoL measured by the SF-36 and its association with depressive symptoms assessed by the PHQ-9 scale and cognitive impairment measured by the MoCA scale in 65 Romanian patients with isolated dystonia. This is the first published study that analysed the association between health-related quality of life and non-motor symptoms in Romanian patients with dystonia. To our knowledge, few studies have jointly analysed non-motor symptoms (depression and cognitive function) and their relationships with HRQoL measured by the SF-36 instrument in patients with dystonia.

In line with review articles and previous studies [6,7,8,42,43], our results showed that depressive symptoms and cognitive impairment were significantly associated with HRQoL and were important predictors of HRQoL among patients with dystonia. Patients with fewer depressive symptoms had higher PCS and MCS scores. Similarly, better cognitive performance correlated with higher PCS and MCS. Results of multiple regression analysis showed that the two non-motor symptoms explained approximately 38% of the variance in PCS and approximately 58% of the variance in MCS. The higher percentage of explained variance shows that PHQ-9 and MoCA are better predictors of the MCS. However, between these two, we found that depression had a greater impact on HRQoL than cognitive impairment.

In the current study, depression proved to be a major predictor of HRQoL in patients with dystonia. This finding agrees with previous studies conducted in patients with various types of dystonia. A study among 289 patients with cervical dystonia from seven European countries found that depression and anxiety were the strongest predictors of HRQoL [23]. In another study that included 101 Polish and French patients with cervical dystonia, depression was the main predictor of poor HRQoL [44]. Another study reported that depression and anxiety were the most important predictors of poorer HRQoL in 157 Serbian patients with various types of focal dystonia (cervical dystonia, blepharospasm, and writer’s cramp) [45]. Similarly, ref. [20] reported that the most important predictors of HRQoL in patients in the Netherlands with cervical dystonia were severity of depressive symptoms, pain, and disability. A study conducted on Slovak patients with cervical dystonia [21] revealed sleep, depression, and fatigue to be important determinants of poor HRQoL. A regression model with depression (measured using the BDI) and excessive daytime sleepiness (EDS) explained 57.5% of the variation in mental health (MCS).

Furthermore, a study showed that depression was also the main predictor of poor HRQoL in Chinese patients with blepharospasm [46]. Recently, a study among patients with various forms of craniofacial movement disorder found that depression had a greater impact on HRQoL than anxiety [47]. Another recent study reported that depression and fatigue were major determinants of HRQoL in patients with writer’s cramp [48]. A more recent longitudinal study analysing HRQoL data in an international sample of 155 patients with adult-onset isolated dystonia concluded that the most comprehensive predictors of HRQoL in isolated dystonia are symptoms of depression [43].

Our study found a moderate to strong decline in physical health (PCS) and a strong decline in mental health (MCS) with increasing depression levels (PHQ-9). In comparison with non-depressed patients, depressed patients showed poorer HRQoL in all eight SF-36 domains as well as in physical health and in mental health. Similar results were reported by other authors [43,44,45,47,48]. However, in comparison with patients without cognitive impairment, patients with cognitive dysfunction showed poorer HRQoL only in physical health (PCS), physical functioning, and bodily pain. Although not statistically significant, the mean scores of the other domains, including mental health (MCS), were higher (better) in the group of patients without cognitive impairment.

In our study, the scales that measure both positive and negative aspects of well-being (general health, vitality, and mental health) have lower mean scores, in comparison with scales measuring health-related limitations (physical functioning, social functioning, and role emotional), except for physical role and bodily pain. The mean scores were also approximately similar in bodily pain, vitality, and mental health and higher in other SF-36 domains in Polish and French patients with cervical dystonia [44]. Our findings differed more substantially from two studies in China that focused on patients with blepharospasm [46] and writer’s cramp [48]. However, the mean scores of all eight SF-36 domains in our study were considerably lower than those from a recent Italian study with several forms of dystonia [49]. These differences in scores might be due to differences in sample size, age, age at onset, type of dystonia, duration of disease, and socio-economic context.

A comparison of SF-36 scores with normative data of the general population of some countries showed that the patients with dystonia in our study had lower HRQoL scores in all domains and below the European average [50]. Unfortunately, the standards for the SF-36 questionnaire in the Romanian general population do not exist; thus, there was no possibility of comparing the HRQoL of dystonia patients to our standard population. Therefore, we compared our results with the recently published norm of the general population in Hungary [38]. The lowest scores reported by patients with dystonia were in the domains of physical role (51.27 vs. 74.74) and bodily pain (57.92 vs. 76.16), as well as in physical functioning (73.85 vs. 81.72) and mental health (64.18 vs. 69.95), compared with the norm available from the Hungarian general population [38]. In other domains, the mean scores were approximately similar (below five points). The mean PCS and MCS scores were lower in our patients with dystonia: 43.51 vs. 45.61 and 46.22 vs. 50.14, respectively.

Regarding depression, measured in our study using the PHQ-9 scale, 46.2% of patients reported at least mild depressive symptoms (PHQ-9 ≥ 5), and 23.1% reported moderate or more severe depression (PHQ-9 ≥ 10). The prevalence of depression was relatively high and comparable with the results reported in several previous studies in patients with cervical dystonia [18,21,44,51], which emphasize these symptoms as an important factor in the disease process. However, the prevalence of depressive symptoms in our study was higher than the prevalence reported in several studies in patients with cervical dystonia [52,53,54,55], blepharospasm [18,52,55] or in mixed samples of idiopathic dystonia [19,52,56]. However, in our study, prevalence was lower than in another study that found a higher prevalence (56.1%) of depression among patients with craniocervical dystonia [57]. Likewise, a comprehensive systematic review and meta-analysis reported a mean pooled prevalence of depressive symptoms or disorders of 31.5% in cervical dystonia, 29.2% in cranial dystonia, and 30.9% in mixed dystonia populations, based on studies using both rating scales and structured interviews [58]. Their analysis confirms a consistently high prevalence of depression across adult-onset idiopathic dystonia, with moderate heterogeneity driven by the type of screening instrument [58]. Our findings confirm that depression is a common and clinically relevant non-motor symptom in isolated dystonia, consistent with previous literature, despite variation across assessment tools.

Regarding cognitive function, measured in our study using the MoCA scale, cognitive impairment (MoCA < 26) was observed in 35.4% of patients with adult-onset isolated focal dystonia. This prevalence is slightly higher than the 27% reported by Ospina-García et al. in a sample of 44 patients with craniocervical dystonia, although they used a more stringent threshold (MoCA ≤ 18) [57]. Our findings also indicate a higher prevalence of cognitive impairment compared to the 13% reported by D’Iorio et al., whose study applied an even stricter adjusted cutoff (<17.2) and found a lower mean MoCA score of 21.3, indicating more severe cognitive impairment in their study group [59].

Additionally, our results are consistent with those of Yilmaz and Bilen, who demonstrated significantly lower MoCA scores in cervical dystonia patients compared to healthy controls (24.23 vs. 26.14), further supporting the presence of cognitive dysfunction in this patient population [60]. Overall, these findings reinforce the concept that cognitive impairment is a relevant non-motor symptom in dystonia, in line with our observations. On the other hand, the MoCA scores in our study are lower compared to those reported in another study [61]. The differences in MoCA values across studies can be explained using different cutoffs [59,61].

In our analysis, cognitive impairment was more prevalent among female patients (40.4%) than male patients (15.4%), with mean MoCA scores of 25.83 in female patients and 27.54 in male patients (*p* = 0.115). This observation is indirectly supported by several studies [54,59].

Furthermore, no statistically significant associations were observed between cognitive impairment and other demographic or clinical variables in our study. These findings are consistent with previous studies [54,59,60,61,62]. This reinforces the view that cognitive impairment in dystonia likely reflects intrinsic non-motor dysfunction rather than secondary effects of clinical or demographic factors.

In our sample, a significant negative correlation was found between cognitive function and depression severity (r = −0.25, *p* = 0.041), suggesting that greater depressive symptoms are modestly associated with lower cognitive function. Similarly, a nested case-control analysis identified an increased frequency of depression and cognitive dysfunction in patients with facial dystonia, particularly blepharospasm, based on PHQ-9 and MoCA evaluations [51]. In a cross-sectional pilot study, nearly half of the patients with focal dystonia were found to have depressive symptoms, and a similar proportion showed cognitive impairment, both linked to more negative self-assessments of disease severity [26].

These findings are in line with prior research, suggesting a significant association between cognitive impairment and depressive symptoms in dystonia [12,63]. Similar patterns have also been reported in other neurodegenerative disorders, such as Parkinson’s disease, where co-occurrence of cognitive deficits and depressive symptoms is well-documented [64,65].

However, the relationship between these non-motor symptoms in dystonia is not uniformly observed. For instance, Ospina-García et al. (2020) found no significant association between cognitive impairment and depression in patients with craniocervical dystonia [57]. These discrepancies may reflect heterogeneity in dystonia phenotypes, assessment tools, or sample characteristics.

This study provides valuable insights into the relationship between non-motor symptoms and health-related quality of life (HRQoL) in patients with isolated dystonia. However, several methodological considerations should be acknowledged.

First, the cross-sectional design precludes any inference of causality between HRQoL, cognitive impairment, and depressive symptoms. The observed associations should therefore be interpreted as correlational rather than directional or predictive.

Second, the cohort consisted of 65 patients recruited from a single tertiary care centre who were able to complete extensive assessments during scheduled follow-up visits. This may introduce selection bias, as patients with greater disability or time constraints could be underrepresented. Consequently, the generalizability of these findings to the wider dystonia population may be limited.

Although validated instruments were used and all statistical assumptions were met, no formal a priori power calculation was performed. Nonetheless, the final sample size (n = 65) was consistent with empirical subject-to-variable ratio guidelines for exploratory analyses and allowed for the construction of stable two-predictor linear regression models. These models yielded statistically significant findings for both PCS (adjusted R^2^ = 0.376) and MCS (adjusted R^2^ = 0.584), indicating that a meaningful proportion of the variance in HRQoL was explained. Larger samples in future studies would improve statistical precision and external validity.

In addition, sex, and age, although recorded and analyzed descriptively and in bivariate tests, were excluded from the final multivariable models due to a lack of significant association with outcomes or predictors. Their inclusion did not alter the main effects, and a parsimonious model was preferred to maintain stability, consistent with recommendations for exploratory studies.

Another limitation is the absence of data on current antidepressant use and lifetime history of depression. These factors could have confounded PHQ-9 scores and may partly obscure associations with HRQoL. Future studies should incorporate such variables to improve the accuracy of mood-related assessments in dystonia.

Despite these limitations, this study contributes meaningful evidence on the impact of non-motor symptoms in dystonia. The findings support the routine inclusion of cognitive and mood assessments in clinical practice, particularly in settings where such evaluations are not yet standard.

## 5. Conclusions

This study confirmed that non-motor symptoms have significance when assessing the impact of the disease by examining the relationship between depression, cognitive impairment, and health-related quality of life in patients with isolated dystonia. We observed that lower HRQoL was substantially correlated with both cognitive deficits and depressive symptoms, with depression having a more obvious negative impact than cognitive impairment. This is one of the first studies to examine these factors together in a Romanian dystonia cohort, providing new proof that patients’ well-being is significantly impacted by both cognitive and psychiatric comorbidities. Our findings are distinctive because they reveal that depression is the most influential independent predictor of lower quality of life in dystonia, even more significant than objective cognitive status. This supports findings from other populations and applies them to an Eastern European clinical setting.

Clinically, these outcomes highlight how important it is to regularly screen for and treat depression and cognitive dysfunction in the treatment of dystonia. Regular neurology visits that include standardized tests (such as the PHQ-9 and MoCA) may help identify patients who are at risk. Prompt interventions (including antidepressant therapy, cognitive rehabilitation, or counselling) may enhance patients’ mood and cognitive function as well as their general quality of life. Our results encourage a more patient-centred, multidisciplinary approach to isolated dystonia, where addressing non-motor symptoms is acknowledged as important for improving results. This viewpoint may help to improve current practice guidelines.

When interpreting the findings, it is important to acknowledge several of the study’s limitations. First, although associations are evident, we are unable to determine whether depression causes lower HRQoL or vice versa due to the cross-sectional design, which prevents any causal conclusions. Second, the sample was small and only included patients from one tertiary facility, which could limit the findings’ applicability to all dystonia patients and introduce selection bias (patients who could attend follow-ups). Additionally, some confounders were not taken into consideration (e.g., sex, age), which may affect depression scores and quality of life.

To confirm these associations over time, future studies should use longitudinal designs and larger, multi-centre cohorts. Such research could investigate other non-motor factors (such as anxiety, pain, or sleep disturbances) in addition to cognitive changes, and it could look at whether successfully treating depression in dystonia patients results in noticeable improvements in HRQoL. Our study concludes that non-motor symptoms, especially depression, are important factors that influence quality of life in people with isolated dystonia. Moving forward, professionals should prioritize addressing these symptoms as part of comprehensive dystonia management, as it has the potential to significantly improve patient well-being.

## Figures and Tables

**Table 1:** Demographic and clinical characteristics.

Characteristics	Mean (SD) or n (%)
Sex, n (%)	
Female	52 (80)
Male	13 (20)
Age at survey (years), mean (SD)	56.57 (14.34)
Age at survey groups, n (%)	
≤44 years	16 (24.6)
45–54 years	9 (13.8)
55–64 years	19 (29.2)
65+ years	21 (32.3)
Education (years), mean (SD)	12.86 (2.28)
Education level, n (%)	
≤12 years	43 (66.2)
>12 years	22 (33.8)
Age at onset (years), mean (SD)	48.34 (13.05)
Age at onset groups, n (%)	
≤40 years	19 (29.2)
>40 years	46 (70.8)
Body distribution, n (%)	
Focal	45 (69.2)
Segmental	20 (30.8)
Type of dystonia, n (%)	
Cervical dystonia	30 (46.2)
Craniofacial dystonia	33 (50.7)
Other	2 (3.1)
Disease duration (years), mean (SD)	8.23 (7.41)
Disease duration groups, n (%)	
≤10 years	44 (67.7)
>10 years	21 (32.3)

Abbreviations: SD = standard deviation; n = number of patients;

**Table 2:** Descriptive statistics for SF-36 scale score and summary measures (n = 65).

Variable	Mean	SD	Mdn	IQR	Min	Max
Physical functioning (PF)	73.85	24.12	85	55–95	0	100
Role physical (RP)	51.27	45.13	50	0–100	0	100
Bodily pain (BP)	57.92	32.09	60	32.5–90	0	100
General health (GH)	60.32	21.40	60	42.5–75	20	100
Vitality (VT)	58.69	23.70	65	47.5–75	0	95
Social functioning (SF)	84.28	23.92	95	87.5–100	0	100
Role emotional (RE)	77.44	40.44	100	66.6–100	0	100
Mental health (MH)	64.19	20.66	64	48–80	12	96
Physical component summary (PCS)	43.52	10.56	46.6	37.2–52.7	18.7	58.3
Mental component summary (MCS)	46.22	10.37	47.9	38.3–55.0	21.8	61.9

Abbreviations: n = number of patients; SD = standard deviation; Mdn = median; IQR = interquartile range.

**Table 3:** Correlations among SF-36 scales and component scores.

	RP	BP	GH	VT	SF	RE	MH	PCS	MCS
PF	0.55 **	0.42 **	0.47 **	0.55 **	0.58 **	0.36 **	0.59 **	0.73 **	0.65 **
RP	1	0.50 **	0.22	0.36 **	0.43 **	0.35 **	0.41 **	0.81 **	0.50 **
BP		1	0.43 **	0.44 **	0.42 **	0.31 *	0.31 *	0.77 **	0.50 **
GH			1	0.54 **	0.47 **	0.31 *	0.48 **	0.63 **	0.65 **
VT				1	0.78 **	0.35 **	0.62 **	0.66 **	0.83 **
SF					1	0.61 **	0.74 **	0.65 **	0.87 **
RE						1	0.45 **	0.47 **	0.62 **
MH							1	0.57 **	0.89 **
PCS								1	0.77 **

Abbreviations: PF = physical functioning; RP = physical role; BP = bodily pain; GH = general health; VT = vitality; SF = social functioning; RE = emotional role; MH = mental health; PCS = physical component summary; MCS = mental component summary. * *p* < 0.05. ** *p* < 0.01.

**Table 4:** Bivariate analysis between physical health and demographic and clinical variables.

Variable	n	%	PF	RP	BP	GH	PCS ^b^
Sex							
Female	52	80	69.90 (24.42)	45.34 (44.81)	54.04 (31.85)	59.04 (22.21)	41.80 (10.34)
Male	13	20	89.61 (15.20)	75.00 (39.53)	73.46 (29.20)	65.46 (17.64)	50.34 (8.77)
*p*-value			0.003 ^a^	0.033	0.050	0.337	0.008
Age at survey, years							
≤44	16	24.6	83.12 (20.73)	65.62 (43.66)	70.00 (29.03)	74.44 (17.48)	48.74 (8.45)
45–54	9	13.8	67.78 (23.19)	52.78 (47.51)	35.56 (23.97)	50.00 (22.22)	39.53 (10.15)
55–64	19	29.2	72.89 (23.71)	53.68 (46.78)	65.92 (32.29)	53.68 (20.54)	43.64 (10.94)
65+	21	32.3	70.24 (26.81)	37.50 (42.94)	51.07 (32.30)	60.00 (20.25)	41.13 (10.32)
*p*-value			0.335	0.308	0.028	0.009	0.097
Education, years							
≤12	43	66.2	71.16 (26.25)	48.43 (45.73)	53.84 (31.67)	56.42 (21.38)	42.32 (11.08)
>12	22	33.8	79.09 (18.75)	56.82 (44.44)	65.91 (32.12)	67.95 (19.74)	45.84 (9.25)
*p*-value			0.069 ^a^	0.483	0.153	0.039	0.206
Age at onset, years							
≤40	19	24.6	79.21 (23.64)	60.53 (45.88)	67.76 (29.88)	71.63 (19.00)	47.13 (10.14)
40+	46	75.4	71.63 (24.22)	47.45 (44.76)	53.86 (32.40)	55.65 (20.75)	42.02 (10.47)
*p*-value			0.252	0.291	0.113	0.005	0.076
Body distribution							
focal	46	70.8	78.04 (21.69)	59.13 (42.70)	60.98 (31.16)	64.04 (19.80)	45.57 (9.48)
segmental	19	29.2	63.68 (27.17)	32.24 (46.27)	50.53 (33.95)	51.32 (22.96)	38.54 (11.62)
*p*-value			0.028	0.028	0.235	0.028	0.014
Disease duration							
≤10 years	44	67.7	79.10 (19.57)	60.23 (42.56)	61.42 (29.73)	62.41 (20.82)	45.83 (8.61)
>10 years	21	32.3	62.86 (29.18)	32.50 (45.55)	50.59 (36.21)	55.95 (22.45)	38.67 (12.69)
*p*-value			0.040 ^a^	0.019	0.206	0.259	0.046 ^a^

Abbreviations: PF = physical functioning; RP = physical role; BP = bodily pain; GH = general health; PCS = physical component summary. ^a^ non-parametric tests were applied (Mann–Whitney U test or Kruskal–Wallis H test); ^b^ T-scores (mean = 50, SD = 10) calculated against the general US population.

**Table 5:** Bivariate analysis between physical health and demographic and clinical variables.

Variable	n	%	VT	SF	RE	MH	MCS ^b^
Sex							
Female	52	80	55.58 (24.19)	81.24 (25.79)	75.00 (42.72)	62.00 (20.92)	44.86 (10.65)
Male	13	20	71.15 (17.34)	96.42 (4.85)	87.18 (28.99)	72.92 (17.67)	51.68 (7.19)
*p*-value			0.033	0.058 ^a^	0.593 ^a^	0.088	0.036 ^a^
Age at survey, years							
≤44	16	24.6	63.44 (20.22)	90.75 (15.64)	83.33 (36.51)	69.00 (21.69)	49.61 (8.49)
45–54	9	13.8	52.78 (25.14)	84.56 (22.83)	62.96 (48.43)	62.22 (21.69)	43.29 (10.46)
55–64	19	29.2	58.16 (23.35)	81.00 (25.72)	77.19 (41.65)	61.47 (22.87)	45.23 (11.32)
65+	21	32.3	58.09 (26.71)	82.19 (28.19)	79.36 (40.11)	63.81 (20.73)	47.79 (10.81)
*p*-value			0.756	0.867 ^a^	0.682	0.742	0.461
Education, years							
≤12	43	66.2	59.77 (23.32)	84.51 (23.39)	75.97 (41.36)	63.81 (19.08)	45.94 (9.92)
>12	22	33.8	56.59 (24.85)	83.82 (25.47)	80.30 (39.38)	64.91 (23.90)	46.78 (11.42)
*p*-value			0.613	0.913	0.686	0.842	0.760
Age at onset, years							
≤40	19	24.6	60.26 (22.08)	88.39 (18.61)	80.71 (38.99)	68.00 (20.48)	48.46 (9.35)
40+	46	75.4	58.04 (24.55)	82.58 (25.79)	76.09 (41.37)	62.61 (20.74)	45.29 (10.73)
*p*-value			0.734	0.881 ^a^	0.679	0.343	0.267
Body distribution							
focal	46	70.8	61.96 (22.05)	86.77 (23.33)	81.16 (36.95)	66.61 (20.54)	47.82 (9.81)
segmental	19	29.2	51.25 (25.64)	78.24 (24.87)	68.42 (47.76)	58.32 (20.28)	42.34 (10.94)
*p*-value			0.084	0.193	0.251 ^a^	0.142	0.052
Disease duration							
≤10 years	44	67.7	62.95 (19.24)	89.69 (17.97)	80.30 (37.57)	67.00 (20.72)	48.09 (9.38)
>10 years	21	32.3	49.76 (29.64)	72.95 (30.61)	71.43 (46.29)	58.29 (19.70)	42.29 (11.46)
*p*-value			0.121 ^a^	0.046 ^a^	0.412	0.112	0.034

Abbreviations: VT = vitality; SF = social functioning; RE = emotional role; MH = mental health; MCS = Mental component summary. ^a^ non-parametric tests were applied (Mann–Whitney U test or Kruskal–Wallis H test); ^b^ T-scores (mean = 50, SD = 10) calculated against the US reference population.

**Table 6:** Descriptive statistics for MoCA and PHQ-9 stratified by sex.

Variable	Total (n = 65)		Female (n = 52)		Male (n = 13)	
	Mean (SD)	Median (IQR)	Mean (SD)	Median (IQR)	Mean (SD)	Median (IQR)
MoCA	26.17 (3.16)	27.00 (25, 28)	25.83 (3.33)	26.00 (25, 28)	27.54 (1.90)	28.00 (27.0, 28.5)
PHQ-9	5.95 (5.79)	4.00 (2, 9)	6.08 (5.70)	4.00 (2, 9)	5.46 (6.38)	3.00 (1.0, 8.5)

Abbreviations: SD = standard deviation; IQR = interquartile range; n = number of patients.

**Table 7:** Frequencies and percentages for MoCA and PHQ-9 category scores.

Variables	Total (n = 65)	Female (n = 52)	Male (n = 13)
MoCA groups, n (%)			
normal (≥26)	42 (64.6)	31 (59.6)	11 (84.6)
abnormal (<26)	23 (35.4)	21 (40.4)	2 (15.4)
PHQ-9 groups, n (%)			
no depression (0–4)	35 (53.8)	27 (51.9)	8 (61.5)
mild (5–9)	15 (23.1)	13 (25.0)	2 (15.4)
moderate (10–14)	8 (12.3)	6 (11.5)	2 (15.4)
mod. severe (15–19)	4 (6.2)	4 (7.7)	0 (0.0)
severe (20–27)	3 (4.6)	2 (3.8)	1 (7.7)

**Table 8:** Correlation between the SF-36 measures and non-motor symptoms.

Variable	PF	RP	BP	GH	VT	SF	RE	MH	PCS	MCS
MoCA	0.317 **	0.340 **	0.352 **	0.122	0.187	0.170	0.153	0.202	0.332 **	0.260 *
PHQ-9	−0.478 **	−0.261 *	−0.334 **	−0.524 **	−0.678 **	−0.692 **	−0.414 **	−0.763 **	−0.515 **	−0.783 **

Abbreviations: PF, physical functioning; RP, physical role; BP, body pain; GH, general health; VT, vitality; SF, social functioning; RE, emotional role; MH, mental health; PCS, Physical Component Summary; MCS, Mental Component Summary; MoCA, Montreal Cognitive Assessment; PHQ-9, Patient Health Questionnaire -9. * *p* < 0.05 ** *p* < 0.01.

**Table 9:** Results of the multiple regression analysis.

SF-36 Summary Measures	Predictors	R^2^	Adjusted R^2^	B	β	*p*-Value	Confidence Interval	
							Lower	Upper
PCS	MoCA	0.395	0.376	1.115	0.334	0.002	0.442	1.788
	PHQ-9			−0.859	−0.471	<0.001	−1.226	−0.492
MCS	MoCA	0.597	0.584	0.542	0.165	0.049	0.003	1.082
	PHQ-9			−1.295	−0.723	<0.001	−1.589	−1.000

Abbreviations: PCS, Physical Component Summary; MCS, Mental Component Summary; MoCA, Montreal Cognitive Assessment; PHQ-9, Patient Health Questionnaire -9; R^2^, explained variance; B, unstandardized coefficients; β, standardized coefficients.

**Table 10:** Relationship between SF-36 scales and non-motor symptom scoring categories.

Physical Health						
Variable	n (%)	PF	RP	BP	GH	PCS ^a^
MoCA groups						
Normal (≥26)	42 (64.6)	76.90 (22.82)	61.19 (42.95)	65.12 (29.62)	59.79 (21.71)	45.49 (9.74)
Abnormal (<26)	23 (35.4)	68.26 (25.92)	33.15 (44.21)	44.78 (32.89)	61.30 (21.28)	39.90 (11.24)
*p*-value		0.169	0.015	0.013	0.787	0.040
PHQ-9 groups						
No depression (0–4)	35 (53.8)	83.57 (17.72)	61.79 (43.61)	65.93 (29.55)	70.86 (48.03)	48.37 (7.41)
Depressed (5–27)	30 (46.2)	62.50 (25.86)	39.00 (44.44)	48.58 (32.88)	48.03 (19.14)	37.85 (10.96)
*p*-value		<0.001	0.041	0.029	<0.001	<0.001 ^b^
**Mental Health**						
**Variable**	**n (%)**	**VT**	**SF**	**RE**	**MH**	**MCS ^a^**
MoCA groups						
Normal (≥26)	42 (64.6)	60.48 (22.38)	84.93 (24.88)	81.75 (36.22)	65.05 (20.89)	47.12 (9.93)
Abnormal (<26)	23 (35.4)	55.43 (26.15)	83.09 (22.54)	69.56 (47.05)	62.61 (20.59)	44.58 (11.18)
*p*-value		0.417	0.769	0.411	0.653	0.349
PHQ-9 groups						
No depression (0–4)	35 (53.8)	71.57 (16.48)	95.47 (11.25)	92.38 (21.52)	76.34 (13.83)	52.70 (5.76)
Depressed (5–27)	30 (46.2)	43.67 (22.09)	71.22 (28.04)	60.00 (48.83)	50.00 (18.19)	38.66 (9.42)
*p*-value		<0.001 ^b^	<0.001 ^b^	0.002 ^b^	<0.001	<0.001 ^b^

Abbreviations: PF, physical functioning; RP, physical role; BP, body pain; GH, general health; VT, vitality; SF, social functioning; RE, emotional role; MH, mental health; PCS, Physical Component Summary; MCS, Mental Component Summary; n number; values for the eight domains and summary measures are mean (M) and standard deviation (SD). ^a^ T-scores (mean = 50, SD = 10) calculated against the US reference population. ^b^ Mann–Whitney U test.

## Data Availability

The datasets presented in this article are not readily available because the authors do not own the database used in the presented statistics. They requested access to the database from the owner, the institution that conducted the study. The owner decided to keep the database private.

## Abbreviations

The following abbreviations are used in this manuscript:

BDI	Beck Depression Inventory
BP	Bodily pain
GH	General health perceptions
HRQoL	Health-Related Quality of Life
IQR	Interquartile Range
MCS	Mental Component Summary
Mdn	Median
MH	Mental health
MoCA	Montreal Cognitive Assessment
NMS	Non-motor symptoms
PCS	Physical Component Summary
PF	Physical functioning
PHQ-9	Patient Health Questionnaire-9
RP	Physical role (limitations due to physical problems)
SD	Standard deviation
SF	Social functioning
SF-36	Short Form-36 Health Survey
VT	Vitality
